# Nanopore sequencing in microgravity

**DOI:** 10.1038/npjmgrav.2016.35

**Published:** 2016-10-20

**Authors:** Alexa B R McIntyre, Lindsay Rizzardi, Angela M Yu, Noah Alexander, Gail L Rosen, Douglas J Botkin, Sarah E Stahl, Kristen K John, Sarah L Castro-Wallace, Ken McGrath, Aaron S Burton, Andrew P Feinberg, Christopher E Mason

**Affiliations:** 1 Tri-Institutional Training Program in Computational Biology and Medicine, New York, NY, USA; 2 Department of Physiology and Biophysics, Weill Cornell Medical College, New York, NY, USA; 3 Center for Epigenetics, Johns Hopkins University School of Medicine, Baltimore, MD, USA; 4 Department of Electrical and Computer Engineering, Drexel University, Philadelphia, PA, USA; 5 JES Tech, Houston, TX, USA; 6 Exploration Integration and Science Directorate, Astromaterials Research and Exploration Science Division, NASA Johnson Space Center, Houston, TX, USA; 7 NASA Postdoctoral Program, NASA Johnson Space Center, Houston, TX, USA; 8 Biomedical Research and Environmental Sciences Division, NASA Johnson Space Center, Houston, TX, USA; 9 Australian Genome Research Facility, Gehrmann Labs, University of Queensland, St Lucia, QLD, Australia; 10 The HRH Prince Alwaleed Bin Talal Bin Abdulaziz Alsaud Institute for Computational Biomedicine, New York, NY, USA; 11 The Feil Family Brain and Mind Research Institute (BMRI), New York, NY, USA

## Abstract

Rapid DNA sequencing and analysis has been a long-sought goal in remote research and point-of-care medicine. In microgravity, DNA sequencing can facilitate novel astrobiological research and close monitoring of crew health, but spaceflight places stringent restrictions on the mass and volume of instruments, crew operation time, and instrument functionality. The recent emergence of portable, nanopore-based tools with streamlined sample preparation protocols finally enables DNA sequencing on missions in microgravity. As a first step toward sequencing in space and aboard the International Space Station (ISS), we tested the Oxford Nanopore Technologies MinION during a parabolic flight to understand the effects of variable gravity on the instrument and data. In a successful proof-of-principle experiment, we found that the instrument generated DNA reads over the course of the flight, including the first ever sequenced in microgravity, and additional reads measured after the flight concluded its parabolas. Here we detail modifications to the sample-loading procedures to facilitate nanopore sequencing aboard the ISS and in other microgravity environments. We also evaluate existing analysis methods and outline two new approaches, the first based on a wave-fingerprint method and the second on entropy signal mapping. Computationally light analysis methods offer the potential for *in situ* species identification, but are limited by the error profiles (stays, skips, and mismatches) of older nanopore data. Higher accuracies attainable with modified sample processing methods and the latest version of flow cells will further enable the use of nanopore sequencers for diagnostics and research in space.

## Introduction

Remote molecular diagnostics on Earth and in space necessitate portable technologies. In microgravity, microbes show increased virulence,^1,2^ while humans show immune dysregulation.^3,4^ This is a precarious combination aboard confined vessels with no ready access to medical professionals and a limited range of supplies. Sequencing technologies could prove critical for rapid responses to medical infections in space, for instance in deciding whether to use antibiotics and, if so, which ones. Single-molecule methods can also identify modified nucleic acids,^5,6,7,8^ a potentially important aspect to monitoring crew health. Nanopore sequencers, as general current sensing devices, could also assist in the search for extra-terrestrial life by increasing the range of detectable polymers beyond the canonical nucleobases of DNA and RNA.^9,10^

The MinION sequencer from Oxford Nanopore Technologies (ONT) is a small sequencing device (4"×1.5"×1", with a mass of ~100 g) that draws power from and transmits data to a computer through a single USB 3.0 connection.^11^ Libraries consist of double-stranded DNA molecules with a hairpin adapter joining the strands at one end and a motor protein attached to the other end. This structure permits sequencing of both strands of the library templates when the template strand, hairpin adapter, and complementary strand pass through the pore in succession. Consensus information from the “2D” reads produces more accurate base calls than template or complement strands alone.

The nucleotides in the pore at a given time disrupt current flow with a signal specific to their identity. “Events” are called at time points where raw electric current measurements change significantly, which should reflect the entry of a single new nucleotide into the pore. However, the current detection process remains noisy and dependent on reaction conditions like temperature. Past versions of the pipeline from ONT used a hidden Markov model (HMM) algorithm with a Viterbi decoder algorithm by Metrichor to call bases from event data,^12^ but traditional alignment software failed to map most reads;^13,14^ for the newest version of the pore (‘R9’), Metrichor implements a recurrent neural network for improved base calling.

Significant challenges remain in the production and interpretation of nanopore sequencing data because of the high error rates (~15% for older 2D reads^13^). However, the long reads, on the order of several thousands of bases or more, are often sufficient to permit taxonomic classification at the species or genus levels.^15^ Furthermore, researchers using modified sample processing methods have shown accuracies of >95% with the R7 pores and MAP-006 sequencing kits.^16^ In addition, recent data have shown that sequencing yield and quality are likely to improve as the technology develops; the latest pores (R9) give accuracies of 95% for 2D reads and 85% for 1D reads.

Considering the portability of the MinION sequencer and the utility of the resulting data for microbial identification, here we tested the MinION device during a parabolic flight to prepare for a 2016 NASA mission aboard the International Space Station. Although sequencing libraries were constructed on the ground for the present experiments, we posit that library preparation could also be performed in space with the liquid handling procedures described in Ref. 17. We also discuss the performance of several existing methods for taxonomic classification using the data from the microgravity flight and a control experiment performed on the ground, and explore new methods for analysis using event data. These experiments demonstrate for the first time that a nanopore sequencer works in microgravity and continues to function after multiple changes in G-force.

## Results

### In-flight operation

Expanding on a proof-of-principle study regarding liquid handling in microgravity,^17^ we demonstrated the possibility of performing a genomics experiment in space using the MinION sequencer. We prepared two MinION runs using the same sample, the first run over the course of the parabolic flight and the second entirely on the ground using an equally aged flow cell and identical loading procedure. The sample contained equal masses of DNA from three species: Bacteriophage Lambda (cI857*ind* 1 *Sam*7); *Escherichia coli* (K12 MG1655); and mouse (BALB/C female genomic DNA). In a modification to the recommended protocol, we filled syringes (with plastic pipette tips) with 450 μl DNA libraries (see Materials and Methods).

**Figure 1:**
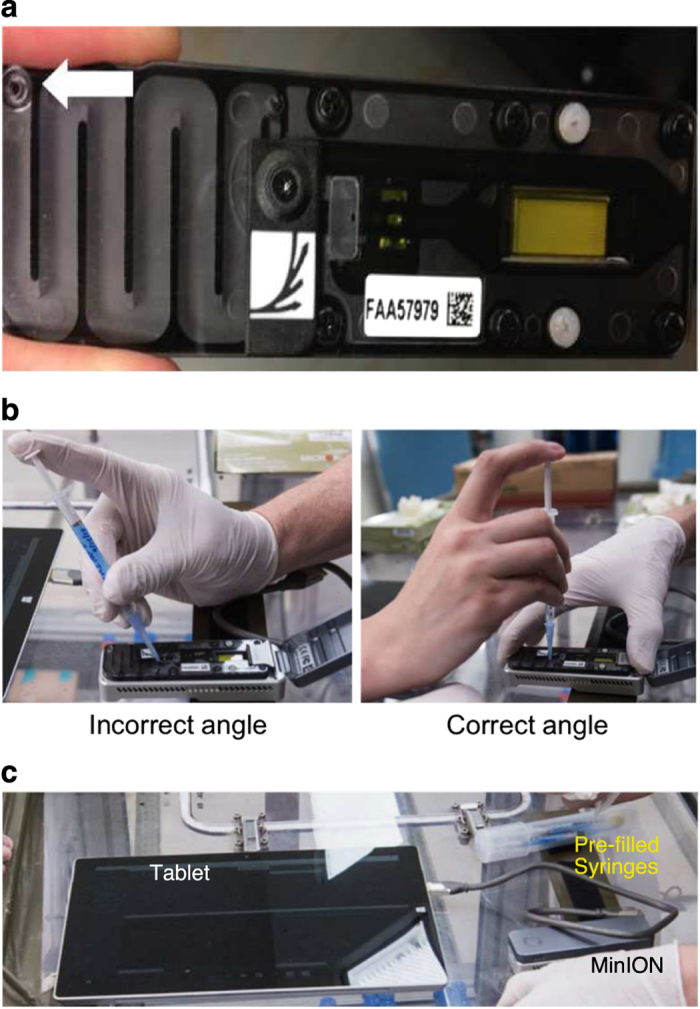
(**a**) The MinION flow cell, which was loaded into the device prior to the parabolic flight. The white arrow marks vents, which leaked during return transport to the Johnson Space Center. (**b**) Loading the library onto the MinION. Angling the pipette perpendicularly to the pore was necessary to avoid introducing air bubbles. (**c**) The MinION setup on the plane. The flow cell was connected to a tablet running Oxford Nanopore Technologies' MinKNOW sequencing software via a USB 3.0 cable. We noted significant glare off of the tablet screen.

Prior to the parabolic flight, we inserted one of the flow cells into the MinION (Figure 1a). During the microgravity portion of a parabola, we removed an air bubble from the input pore of the flow cell using an empty syringe, and then loaded the DNA library mixture (Supplementary Video 1). We did not observe any bubbles in the flow cell pre-flight, and we speculate that gravity or pressure changes may have contributed to the formation of the bubble. To prevent the introduction of a new air bubble, the library mixture was pushed to the end of the tip before engaging with the sample-loading pore. It was critical when removing the air bubble and loading the DNA library to keep the pipette tip firmly engaged and exactly perpendicular to the sample-loading pore to create a seal (Figure 1b). After loading the library, we connected the MinION device via a USB 3.0 cable to a Microsoft Surface Pro 3 (Houston, TX, USA) tablet running the sequencing software (Figure 1c).

We initiated sequencing after sample loading while there were still ten parabolas remaining in the flight (~1 min each, including 30 s of microgravity) and continued sequencing through transport back to Johnson Space Center (Supplementary Figure 1). After halting and opening the sequencer, we observed that the flow cell had leaked from a vent (Figure 1a, arrow) likely due to being tilted vertically during transport to the Johnson Space Center. We did not observe any fluid leaks during parabolic flight.

A first step when initiating a MinION run is to scan for available pores before sequencing, which we did during the parabolic flight. We observed that the number of available nanopores for sequencing during the parabolic flights (*n*=16) was much lower than the maximum of 2,048 pores (512 channels with four pores each). We have observed wide variation in performance over many ground runs with older flow cell and kit versions, including the MAP-005 kit used in these experiments, and thus normal flow cell variation could account for low pore activity on a flow cell. In addition, flow cells are optimally used within 8 weeks of their receipt, whereas the flow cells used for the parabolic flight and ground control were ~12 weeks old. Nonetheless, enough of the pores were active to generate data (below). Finally, we did not perform a pre-flight scan of available pores for comparison: a key quality control step we are implementing for all flow cells we send to the ISS. Our QC runs on new R7 flow cells indicate those we sent to the ISS will likely have 1,000–1,500 available pores (Supplementary Table 1).

### Further controls

To understand the effects of launch on the flow cells, we performed launch vibration testing on a fresh flow cell (Supplementary Table 2). The careful packing of the flow cells inside bubble wrap within a cargo transfer bag significantly reduced the vibrational forces they experienced, to a maximum of 1.7 *g*. After three intervals simulating movement during launch, most (~70%) of the original pores remained active (Supplementary Table 3), indicating that pores will likely survive travel to the ISS for current and future missions. Also, we conducted five additional ground experiments with R7 flow cells after the parabolic flight to refine the protocol before launch to the ISS and define normal flow cell range (Supplementary Table 1, Supplementary Figure 3).

### MinION data analysis

Despite the technical issues described above, the parabolic flight experiment produced three template strand reads from two MinION channels. Comparing the flight log to the sequencer timestamps, we found that the sequencer generated the longest read in microgravity, and another two after parabolas had concluded (Supplementary Figure 1). Thus, we confirmed that the instrument is capable of producing data in microgravity and even after many gravity transitions. The ground control experiment produced 1,737 template strand (1D) reads from 261 channels, as well as 1,012 complement strand reads and 196 2D pass-filter reads, in ~3 h. For direct comparisons between the experiments, we examined only the template strands of the ground and additional control data unless otherwise noted.

**Figure 2:**
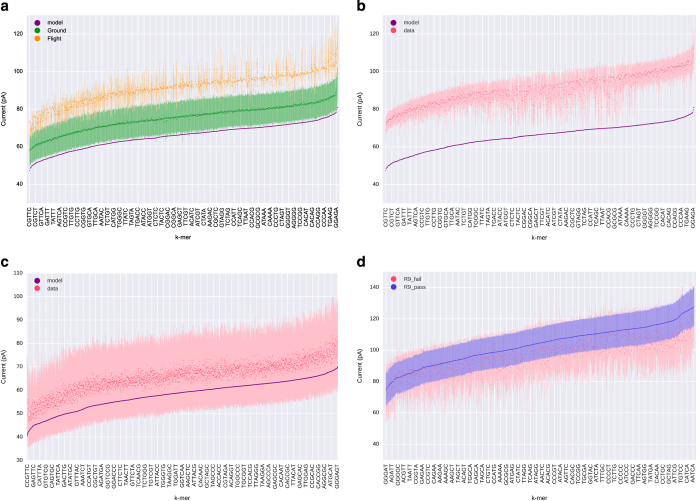
(**a**) Current distributions by 5-mer for flight data, ground data, with k-mers ordered by expected means stored in Metrichor's Hidden Markov Model. S.d. of the mean were calculated across reads for the flight and ground data. (**b**, **c**) The maximum deviations from the models we observed among seven runs with kit version SQK-MAP-005, and five runs with the SQK-MAP-006 kit, moving to a 6-mer model. (**d**) Current distributions for a run with the newest (R9) version of the nanopore. For this version, Metrichor does not provide a model, using a recurrent neural network to base call reads, therefore we compared ‘pass’ (average quality>9) and ‘fail’ reads, ordering k-mers by their mean across pass reads.

The flight data exhibited increased currents across k-mers, with a median of 91.77 pA across reads, whereas the ground data exhibited a median of 74.8 pA (Figure 2a). These data represent mean shifts from the median currents stored in the HMM of 32.1 and 10.8 pA respectively. Both data sets still produced roughly the correct distribution of amperages across different k-mers. Among experiments with older flow cell versions, we had observed similar shifts in current distributions (Figure 2b, Supplementary Figure 2), which may relate to reaction conditions such as temperature.^7^ Shifts of this magnitude did not occur for our additional controls with R7 flow cells (Figure 2c, Supplementary Figure 3). We also see some variation in current levels between experiments with the most recent R9 flow cells (Figure 2d, Supplementary Figure 4), and as such, these data suggest that variation is inherent to flow cells.

**Figure 3:**
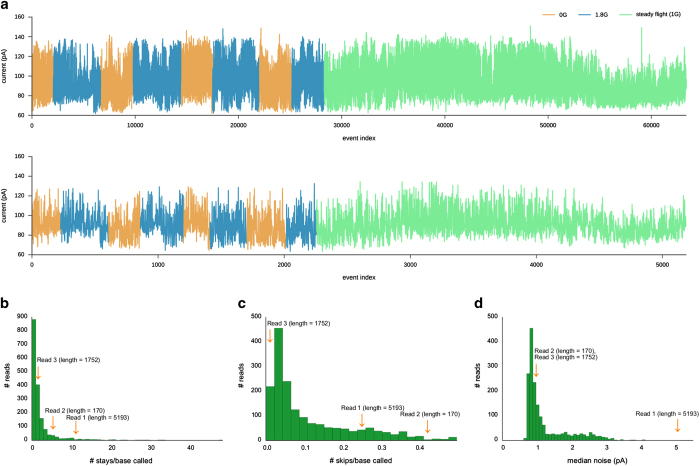
(**a**) The single microgravity flight read, with events separated according to the gravitational force at the time of their initiation. The plot above shows all events of the read, the plot below only those associated with new k-mers after base calling, removing stays and accounting for skips in the index. Currents associated with microgravity showed significant but negligible differences with those associated with 1.8 *g* (*D*=0.08, *P*=1.2×10^−41^) and steady flight (*D*=0.05, *P*=6.2×10^−17^), according to the Kolmogorov–Smirnov test. However, the differences likely represent drifts in current levels over the run, as the first two microgravity segments also significantly differed from the last two (*D*=0.15, *P*=1.0×10^−48^), and the two post-microgravity reads also significantly differed (*D*=0.4, *P*=1.9×10^−139^). (**b**–**d**) Histograms of the number of stays per base, number of skips per base called, and noise for ground data reads. The orange arrows mark the approximate positions of the flight data reads. Median noise is calculated by MinKNOW for each read.

For the read produced in microgravity, we used the time of each parabola stored in the flight log to divide events by gravitational condition. Differences in current level are small as the gravitational conditions change across the read, and may represent drift over the run (Figure 3a). We also assessed various measures of read quality. The ONT event-calling software (MinKNOW) defines “events” at current changes large enough to suggest that a new base has entered the pore. The base-calling algorithm by Metrichor then determines how the sequence of these events should translate to the sequence of nucleotide bases. MinKNOW produced three signals (977, 3,710, and 63,362 events in length) from the flight run, but base calling reduced these signals to 170, 1,752, and 5,193 bases, respectively. The ratio of the number of events to the number of bases reveals a high proportion of “stays”, where an event does not correspond to a new k-mer in the predicted sequence, in the flight data, particularly in the final read. The average number of stays per base called was higher in the flight data reads with a mean of 5.97, as compared with 2.11 for the ground data (Figure 3b). “Skips” in the signal, bases predicted that do not correspond to events, occurred at a much lower rate than stays in both data sets, but still higher in the flight data, with a mean rate of 0.24 skips/base called for the flight data, and 0.11 skips/base called for the ground data (Figure 3c).

The failure to translate over 90% of events to bases in the longest read suggests a high degree of noise. Indeed, the median current noise level as measured by MinKNOW in for the longest flight data read and the only read produced during the parabolas (5.04 pA) was higher than in any of the ground data reads, and the other two flight data reads demonstrated more moderate levels of 0.94 and 0.91 pA, respectively (Figure 3d). For comparison, ground template strands featured a median noise level of 0.92 pA across reads.

### Read classification and alignment

**Figure 4:**
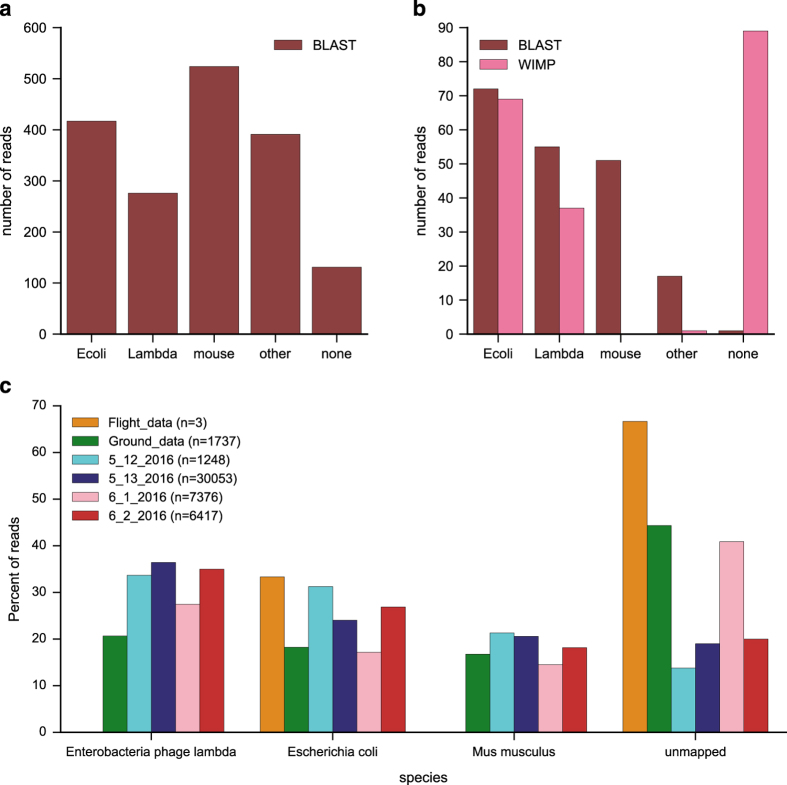
(**a**) Blastn results for template strands from the ground control experiment. BLAST hits were preferentially counted towards the species in the sample even if others scored higher. In the case of ambiguity between multiple sample species, only the highest scoring was considered a hit. (**b**) A comparison of BLAST and Oxford Nanopore Technologies’ WIMP using the 2D reads from the ground experiment. We ran both bacteria and virus versions of WIMP. Here we include any read mapping to *Escherichia coli* or a strain thereof as *Ecoli*, and any read mapping to Lambdalikevirus as Lambda, although the algorithm was not able to identify reads beyond the genus level for viruses. (**c**) Classification results for template strand reads from the flight, ground, and later control experiments aligned to a combined Enterobacteria phage lambda, *Escherichia coli*, and *Mus musculus* (GRCm38.p4) genome using GraphMap (https://github.com/isovic/graphmap). We note the number of reads from each experiment as *n*.

We used multiple computational tools to classify the flight and ground data. First, we ran the Basic Local Alignment Search Tool (BLAST) using blastn settings on the base-called reads to evaluate species detection from the mixed sample.^18^ The shortest of the three reads did not map to any species in the sample, whereas the longest aligned to multiple mammalian species including mouse and human, but with only 8% query coverage for the top mouse hit. The medium-length read mapped moderately well to *E. coli*, with 67% identity and 92% query coverage. BLAST results for the template strands were typically poor, with an average identity of 78%, but only 37% query coverage (Figure 4a). Almost a third of reads did not map to any of the three sample species. However, running BLAST on the 2D ground data reads returned 55 reads as Lambda phage, 72 as *Escherichia coli*, 51 as mouse, 17 as exclusively other species, and 1 as none (Figure 4b). The mean identity for 2D hits was 84%, with mean query coverage of 73%.

We then tested the graphical user interface “What’s in my Pot” (WIMP) for the Kraken taxonomic classifier, which uses k-mer alignments to determine sample identities down to the strain level.^15^ The WIMP pipeline attempts to classify only 2D reads that pass a certain quality threshold (defined as a mean base quality>9). We ran bacterial and viral classifications on the ground data to compare with our BLAST results (Figure 4b). WIMP “Bacteria k24” identified 88 reads over its default threshold score, 87 of which were classified as *E. coli*, with 12 of those further identified as particular strains of that species. The final read was classified as *Methanosarcina barkeri str. Fusaro*. WIMP “Viruses k24” classified 37 reads under the genus *Lambdalikevirus*, and was not able to provide any further details. In total, 124 reads were classified as *E. coli* or *Lambdalikeviru*s by the two versions of WIMP, a number consistent with the BLAST results.

For a more targeted approach relevant to samples of known organisms including eukaryotes, we used the nanopore read aligner GraphMap to classify reads.^19^ Control experiments following an optimized protocol (see Materials and Methods) show fewer unmapped reads using GraphMap (Figure 4c). With low query coverage and identity using BLAST, and given WIMP is only currently applicable to 2D reads, we also tested two alternative approaches based on music processing algorithms for classifying unknown samples using the event data.

### Uncovering Nanopore's fingerprints of genomes

Our first approach, titled Uncovering Nanopore's fingerprints of genomes (UNFOG), attempts to construct fingerprints of reference genomes and reads based on their most informative frequencies over time. This employs methods from Shazam, a mobile application that identifies songs based on short audio clips of user input.^20^ Shazam first fingerprints the reference collection by pairing peaks from the spectrogram of each song and storing the time between these peaks and the time offset from the beginning of the song. The algorithm then attempts to match similarly constructed fingerprints from the user input. Clips are classified based on the number of fingerprints that match a particular song at a consistent time offset. We converted reference genomes into “event space” using the mean currents for each 5-mer stored in the hidden Markov model (HMM) of Metrichor. As seen in Figure 2a, the amperages associated with various k-mers in our real data followed a similar distribution to the mean currents of the model. As in Shazam, we were able to construct fingerprints using peaks in a spectrogram of the signal (Supplementary Figure 5).

**Figure 5:**
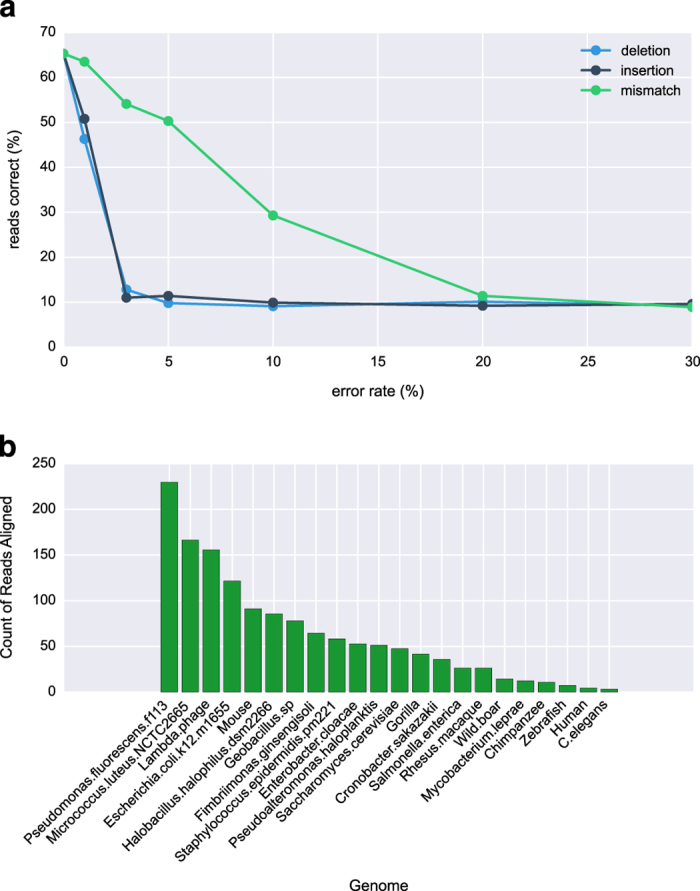
(**a**) Benchmarking results for UNFOG using 5000 base pair fragments of reference genomes. The percent of reads correctly identified as the reference species was 65%, but dropped precipitously as we induced errors into the fragments, with greater tolerance to mismatches than to indels. (**b**) UNFOG results for the ground data. *E. coli* was the top hit, as for BLAST and WIMP using 2D reads, and all three species in the sample were in the top 5 hits among 22 reference species.

We first ran a series of benchmarking tests using a subset of 12 reference bacterial genomes, including highly related species, to determine how well the algorithm is able to classify fragments from a reference genome (*Enterobacter cloacae*, Figure 5a). The best version of UNFOG was able to correctly identify a perfect sample read over 65% of the time and was largely tolerant to up to 10% mismatches. However, the percentage of reads correctly mapped dropped markedly with insertions or deletions in the read. This is similar to what has been found in music identification, where fingerprinting algorithms fail to identify alternative versions of songs due to timing differences, and poses a particular issue for nanopore sequencing. Deletions were found by one study to comprise the largest portion of errors, at roughly double the rate of insertions or mismatches in 2D base-called sequences.^21^

Tests on the flight data failed to classify any of the reads as Lambda phage, *E. coli*, or mouse, identifying one read at *Staphylococcus epidermidis* and a second as either *Halobacillus halophilus* or human. Running UNFOG on ground data revealed more promising results, with all three species present in the top five reference genomes, although *Pseudomonas fluorescens* and *Micrococcus luteus* ranked higher (Figure 5b). We also note that the UNFOG algorithm was able to classify the reads for both flight and ground data in a relatively short time, spanning only 180 s.

### Uncovering Nanopore's signal mapping over genomes

**Figure 6:**
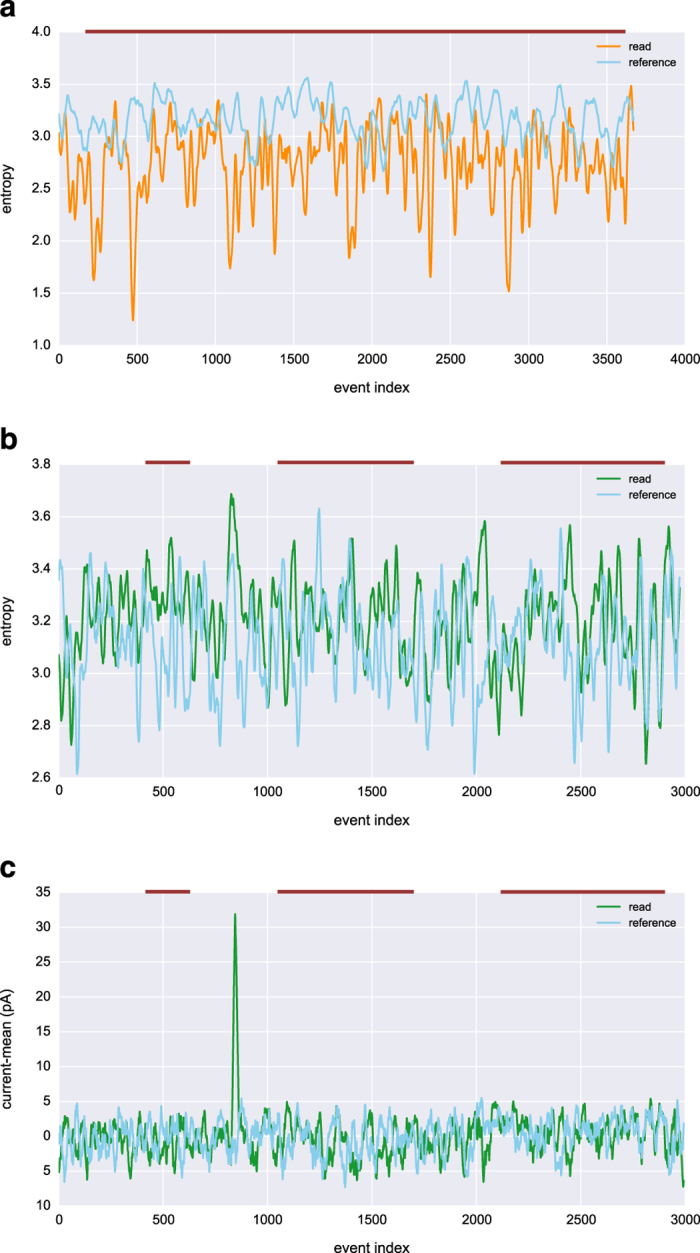
(**a**) The flight data read with the highest mapping score and the subset of *Escherichia coli* genome to which it aligned in entropy space. The reference sequence is shown resampled to match the length of the read, with both signals smoothed by taking the mean over a sliding window of 20. Similarity was found to be 0.01 (*P*=0.5) between the entropy signals using Pearson's correlation. The approximate region of alignment for the BLAST hit is marked as the burgundy line above the plot, although indices do not correspond between event space and the base-called sequence due to the presence of stays and skips in the event space read. (**b**) Entropy signals for a ground data read with a stays per base called ratio of 0.0009, far lower than the mean for these data (2.11). Query coverage for the BLAST alignment of this read to *Lambda phage* was 58%, with 68% identity. The cross correlation between the entropy signals after smoothing was low, *r*=0.15, but positive and significant (*P*=1.25×10^−17^). (**c**) Similarly smoothed event signals for the same ground data read and reference sequence as shown in (**b**), which resulted in lower correlation, *r*=0.04 (*P*=0.06), suggesting that taking the signal entropy may help match event space representations to references.

Our second approach attempts to uncover greater similarity between sequences by converting them to entropy space, estimating the entropy for the signal using a generalized correlation integral approach.^22^ This approach has also been effective in song identification, and has correctly identified the same songs across versions by different artists.^23^ We had previously found that reads showed visually similar entropy patterns to the reference sequences of their BLAST hits. Initial tests with the flight and ground data revealed no such relationship (Figure 6a). However, we suspect that the high rate of stays in most of the reads from these data distorts the signal beyond the limits of this method. Once we reduced our search to reads with stay rates of <0.1 stays/base called, visual similarity was again apparent between the sequences in entropy space, including in areas where BLAST failed to align the sequences (Figure 6b). Sequences that fit the criteria of having a BLAST hit with relatively high query coverage and low stay rate were particularly scarce for this data set, but we had not previously observed this issue with other experiments.

## Discussion

Nanopore sequencing promises to contribute to healthcare in increasingly remote settings, as recently demonstrated during the Ebola outbreak in West Africa.^24^ We show here the beginnings of a new frontier in genomics and genetics, in which humans and robotic rovers could be equipped with DNA sequencers on their travels beyond low Earth orbit. Despite some difficulties in adapting the library loading protocol for the MinION sequencer to the microgravity environment of a parabolic flight and expired flow cells, these data include the first ever DNA sequence generated in microgravity.

We propose several potential modifications to the instrument to facilitate use in reduced gravity. We observed that the attached MinION cover was difficult to control in microgravity when attempting to load the sample (Supplementary Video 2). Though this will be less of an issue in the consistent microgravity environment of the ISS, we recommend a mechanism to keep it in the open position during loading; aboard the ISS, this would most easily be achieved with Velcro. The most important thing to consider when loading a sample is that bubbles are easy to create but difficult to remove. Currently, if the pipette tip is not angled perpendicularly to the loading pore, it is challenging to both remove the initial air bubble and introduce sample into the pore. If the tip is misaligned, instead of removing the initial air bubble from the flow cell, the pipette will draw in ambient air, causing the sample to pool outside the flow cell rather than entering the pore during loading. The successful demonstration of positive displacement pipette use by Rizzardi *et al.* (2016) will enable more robust fluid transfers, and should help resolve these issues by facilitating one-handed operation of the pipette. The crew procedure for use aboard the ISS will emphasize a completely perpendicular loading and firm pressure to completely seal the pore with the pipette tip before loading to prevent pooling on the flow cell surface. Future iterations of nanopore technology may eliminate the need for careful loading procedures. Technologies under development at ONT include the VolTRAX, which aims to automate sample preparation before docking to a sequencer, and the SmidgION, a smaller device that will be compatible with mobile phones.

Future sequencing experiments on the ISS have a clear potential for success. We have optimized the protocol, as described under Materials and Methods for the later experiments with R7 pores, including making the loading procedure for flight a two-step process to cope with limited crew time. Launch vibration testing showed pores will likely survive the trip to the ISS. We also observed leakage from a vent on return to the Johnson Space Center. In the microgravity conditions on the ISS, leaking due to rotation or tilting of the sequencer is extremely unlikely to occur, because surface tension and cohesion should be the dominant forces. On the basis of our observations, the primary issue with the parabolic flight run was a low number of available pores, likely related to the age of the flow cell. Although we speculate that the variable gravitational conditions of the flight could create more opportunities for air bubbles to migrate into the nanopores and obstruct them, we did not observe any changes in signal quality across gravitational conditions (0 vs. 1.8 *g*) for the single read generated over the parabolas. The production of reads during and following repeated exposure to increased gravity and microgravity in the parabolic flight experiment suggests that the device will be capable of sequencing on the ISS. As the technology improves, we also expect that bubble formation will be less of an issue. An independent experiment by the Loose Laboratory on the ground reversed the direction of the 1 *g* acting on the flow cell by flipping the sequencer upside-down twenty minutes into a 40-minute amplicon run and did not observe any differences in quality or current shifts, confirming that gravity does not affect nanopore sequencing.^25^ Of greater concern than gravitational effects for space missions is device stability over extended missions with exposure to higher levels of radiation.^9^ Using flow cells beyond their optimal use period for this experiment illustrated the potential issues with bringing sequencers on long-term mission (e.g., a Mars mission), but also show that even expired reagents can produce data in microgravity. Further development of solid-state nanopores may be necessary for many applications in space research, since protein pores are sensitive to degradation.

In terms of data analysis, for low yield, low-quality runs, the WIMP pipeline from Metrichor is not ideal, as it classifies only 2D reads above a quality threshold. With particularly noisy data, BLAST can also fail to align a majority of reads. Although BLAST was usually able to identify several candidate species for our control data, their scores are not necessarily high or distinct enough to permit accurate identification. Targeted approaches such as GraphMap, though appropriate for our prepared mix of DNA, may not be sufficient for environmental monitoring. While we show that UNFOG has potential in theory as a taxonomic classifier, the nature of errors in nanopore sequencing currently limit its application. An entropy-based solution may be capable of greater accuracy at a significant cost in computational time; however, as we demonstrate here, this fails for reads that are extremely stretched out in time with respect to their reference sequences. Several programs attempt to deal with an analogous problem in music, that of “query by humming”, where, far from the exact versions of songs Shazam requires, a user can identify a song by humming a short segment of melody.^26–28^ As the chemistry continues to improve and error rates decrease, we suggest that adapted methods for fingerprinting could allow for rapid metagenomic classifications using future iterations of nanopore sequencing technology. The greatest advantage to fingerprinting would be speed: UNFOG was able to classify the almost 2,000 reads from the ground data in <3 min after the database was built. For customized sequence-query applications, these and other methods could enable onsite genomics in the most remote environments, including space.

## Materials and methods

### Sample preparation and protocol for parabolic flight

Each of the three types of genomic DNA samples was prepared for sequencing according to the procedures specified by ONT for the MAP-005 kit, beginning with 1 μg of each sample of organismal DNA (bacteriophage lambda, *E. coli* and mouse). To facilitate sample loading during the microgravity intervals of the flight, we deviated from the recommended three-step sample-loading procedure, which entails loading 500 μl of running buffer containing fuel mix, waiting 10 min, and loading an additional 500 μl of buffer and fuel mix with a 10 min wait, followed by loading 150 μl containing running buffer, fuel mix and the sequencing library mix. Instead, we pre-loaded syringes for both the flight and ground control, each containing 450 μl volume of running buffer and fuel mix with 6 μl of pre-sequencing mix from each organism. The library and pre-loaded syringes were prepared two days prior to flight, stored at −20 °C until the day of the flight, and stowed at ambient temperature aboard the plane prior to loading the flow cell.

### R7, SQK-MAP-006 experiments

Genomic DNA samples were sheared individually and then pooled in equal concentrations targeting a concentration between 1.5 and 2 μg DNA (Figures 2c and 4c, Supplementary Figure 3). The DNA was prepared for sequencing according to the procedure specified by ONT for the MAP-006 kit. After library preparation, an aliquot of sample containing 24–84 ng of DNA was diluted with running buffer, fuel mix, and water to the specified concentrations in a total volume of 450 μl. These samples were frozen kept at −80 °C until immediately prior to loading. A 250 μl aliquot of the sample was loaded onto a R7 MinION (Oxford Science Park, Oxford, UK) flow cell. After a 10-min wait the remaining 200 μl of sample was loaded and sequencing was initiated.

### R7, SQK-MAP-005 additional data

A pool of five bacterial genomes was prepared for sequencing via the Oxford Nanopore MinION using the ‘SQK-MAP-005’ library preparation guidelines and reagents (Figure 2b, Supplementary Figure 2). Genomic DNA was analyzed for quality using the Life Technologies Qubit dsDNA BR assay (Carlsbad, CA, USA; PN# Q32850) and Agilent 2200 Tapestation genomic DNA assay (Santa Clara, CA, USA; PN# 5067–5365). New England BioLabs preCR (Ipswitch, MA, USA, PN# M0309S) was used to repair potential DNA damage prior to library preparation. All libraries were sequenced using MinION version 7.3 flow cells.

### R9 data

The DNA sample (metagenomics gDNA from Lake Hillier XMP project) was sheared to 10 kb size using a Covaris G-tube (Figure 2d, Supplementary Figure 4). DNA was then prepared using the Oxford Nanopore R9 protocol (version NSK-007), including FFPE repair, end repair and dA-tailing, adapter ligation and tethering, and Streptavidin recovery. The prepared library was run on an R9 MinION flowcell using MinKNOW version 0.51.1.66 with the “NC_48hr_Sequencing_Run_FLO_Min104.py” protocol. The raw data were called with Metrichor using “2D Basecalling RNN for SQK-NSK007” version 1.99 (Oxford Science Park, Oxford, UK).

### BLAST

Nucleotide-Nucleotide BLAST 2.2.29+ was run using default settings for “somewhat similar sequences,” connecting to NCBI’s most recent nt database (as of 1 November 2015). If there were hits for multiple sample species only the highest scoring was considered. Blast hits were preferentially counted towards the known sample species. Query cover was calculated for the counted hit by taking dividing the length of the primary alignment by that of the query sequence. This differed at times from the query coverage calculated using the online version of BLAST, which is able to calculate over an aggregation of compatible aligning regions, and thus found a query coverage of 26% for the longest flight data read in its alignment to mouse. Identity was as provided by the BLAST alignment report.

### WIMP

Base-calling and species classification were performed using the WIMP Bacteria k24 for SQK-MAP005 (version 1.27) and WIMP Viruses k24 for SQK-MAP005 (version 1.27, Oxford Science Park, Oxford, UK) pipelines. Species counts were combined for Figure 4b, with reads mapping to the genus Lambdalikevirus considered positive hits for Lambda phage in the absence of species-level classification for the Viruses version. We considered hits only above the default threshold score in the GUI; decreasing that threshold to zero showed more false hits for the Bacteria run and had no effect for the Viruses run.

### GraphMap

We aligned to a combined Enterobacteria lambda phage (NCBI reference sequence NC_001416.1), *E. coli* (NCBI reference sequence NC_000913.3), and *Mus musculus* (GRCm38.p4) genome using GraphMap version 0.3.0, with the command “graphmap align -r $ref -d $fi -o $name.sam”, which saves the top result for each read.

### UNFOG

After testing multiple parameter sets, we chose a window size of 128 and overlap of 64 to compute the FFT. We tested several sampling rates; shown are the results using a sampling rate of 10 for the test data and 50 for the real data. Spectrogram peaks over an amplitude of 5 were paired if within 100 of one another on the time axis of the spectrogram for the benchmarking test version, this was changed for the real data version to an amplitude of 20 and time limit of 300 in an attempt to increase specificity. Paired peaks were stored as hashes, along with their offset times for retrieval and comparison.

The benchmarking tests were run using a thousand random 2,500-base fragments of *Enterobacter cloacae* genome joined to their reverse complements to create 2D reads. Errors were induced prior to conversion of the reads from k-mers to currents to mimic possible rates in output base-called sequences. The best version of UNFOG during testing involved storing all instances of a fingerprint across each genome and later removing from consideration any fingerprints that appeared over 50 times in any reference genome. However, this modification did not improve results for the real data (possibly because exact matches are unlikely with high error rates) and significantly increased classification time; therefore, a previous version that saved only the final instance of each fingerprint was used for the real data. In the future, a step discarding the most common fingerprints will likely be incorporated into building the database. For each read, the species with the highest number of matches at a consistent offset time from the beginning of the reference sequence was counted. With both template and complement strands, the same offset time had to be found for both strands for a positive hit. In the case of ambiguous matches, the count for each potential species was increased by 1/(number of matches).

### UNSMOG

Entropy was calculated for each base using a generalized correlation integral approach^22^ with a sliding window of 20 and overlap of 19. We then smoothed the signal by taking the mean over a similarly sized sliding window. These signal were then matched to the raw, entropy-converted nanopore data. Code for UNFOG/UNSMOG (uncovering Nanopore's signal mapping over genomes) is open-source and freely available; posted at http://pbtech-vc.med.cornell.edu/git/mason-lab/unfog.
