# Supplementary material for: Nanopore sequencing in microgravity
Source: NPJ Microgravity. 2016 Oct 20;2:16035–. doi: 10.1038/npjmgrav.2016.35 (PMC5515536; doi:10.1038/npjmgrav.2016.35)
Supplement: Supplementary Information [file npjmgrav201635-s1.pdf]

Nanopore Sequencing in Microgravity
Supplementary Information

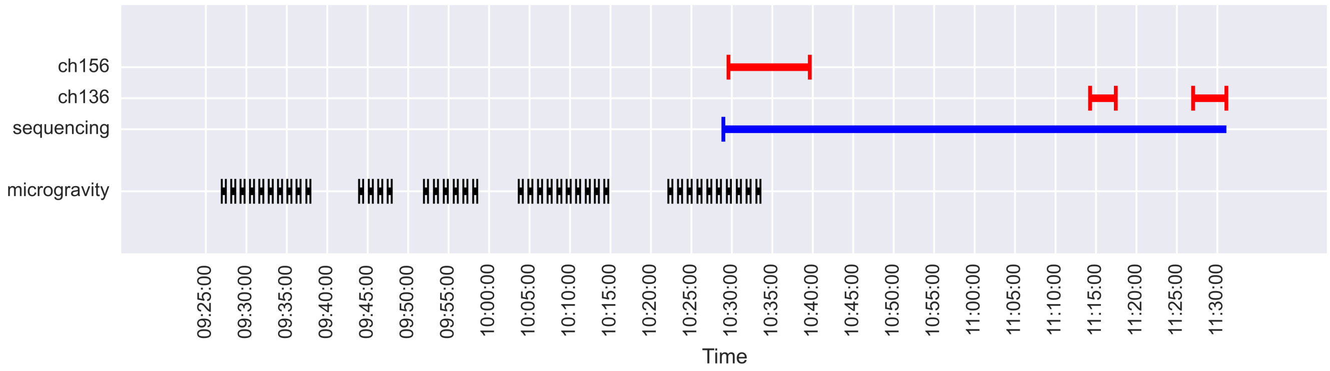

Supplementary Figure 1. Comparison of the timing for periods of microgravity as recorded in the flight log (bottom) with times recorded by the sequencing software for the initiation of the run (blue), and the production of reads by particular channels (red). One read was generated over the last four periods of microgravity.

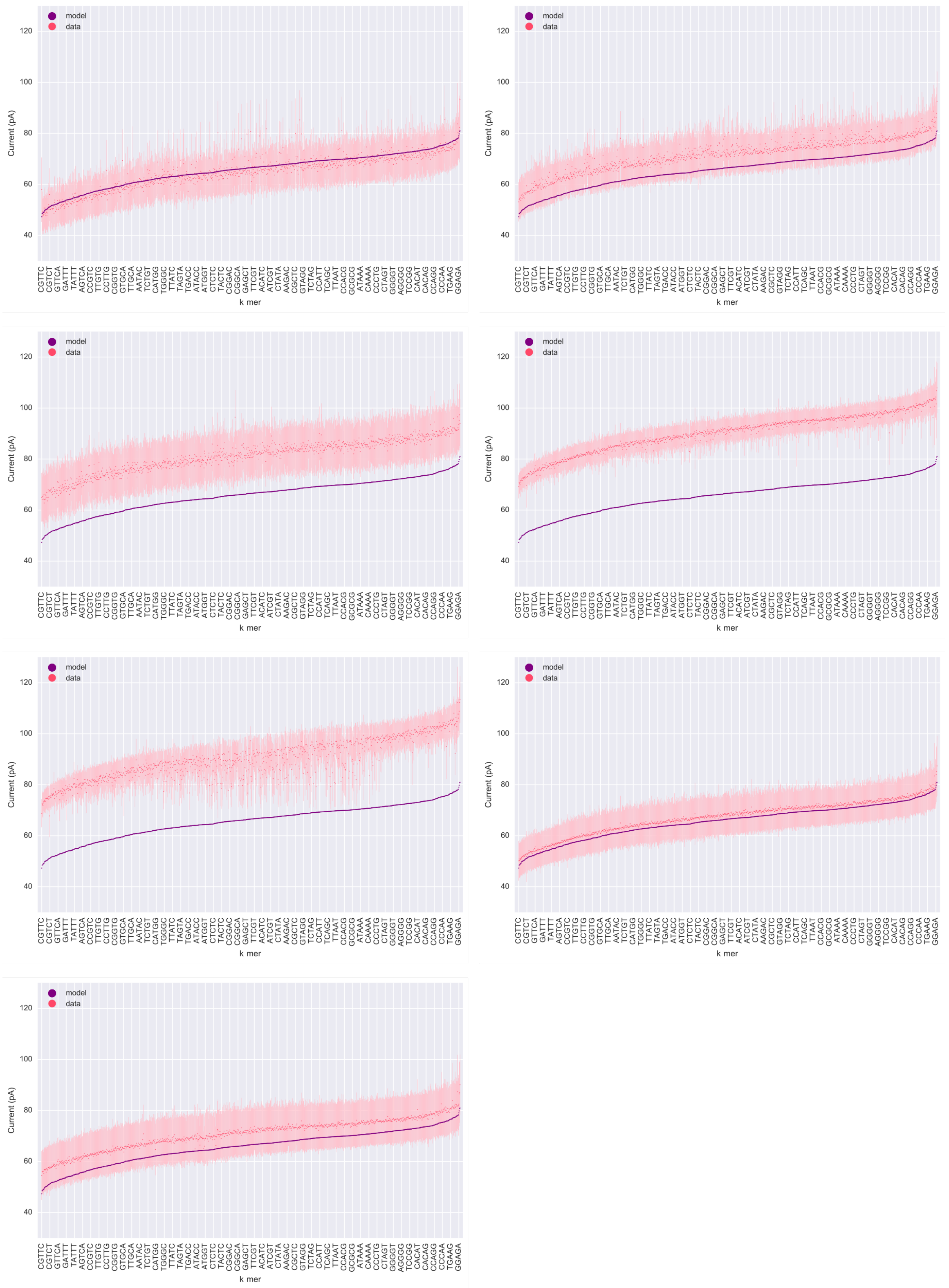

Supplementary Figure 2. Current distributions for nanopore experiments using R7 pores and kit version SQK-MAP-005. The third down on the left corresponds to Figure 2b.

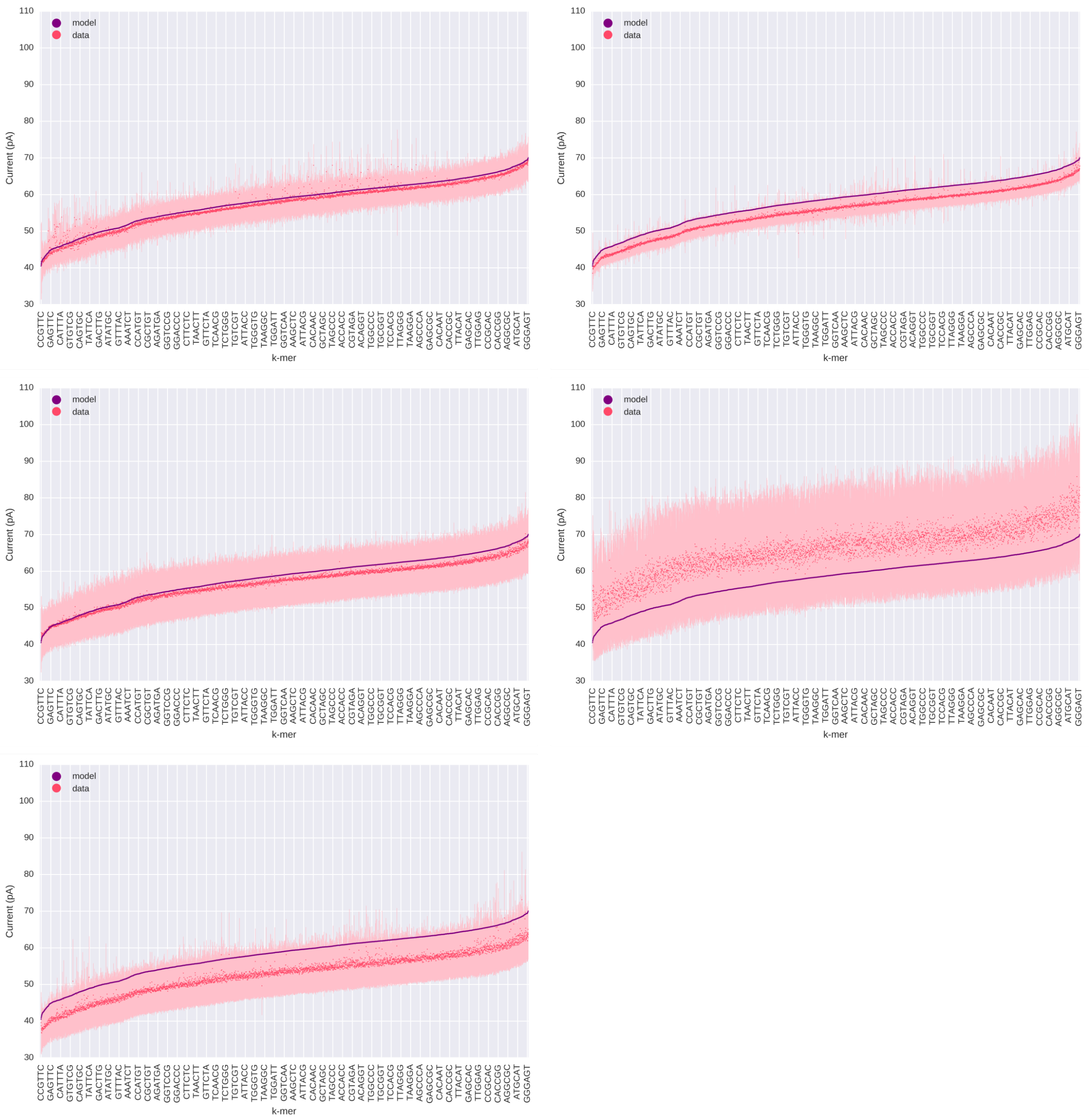

Supplementary Figure 3. Current distributions for experiments using R7 pores and kit version SQK-MAP-006, corresponding, from top to bottom and left to right by row, to an older version of the three species sample preparation, and the experiments labelled 5_12_2016, 5_13_2016, 6_1_2016 (also Figure 2c), and 6_2_2016 in Figure 4c.

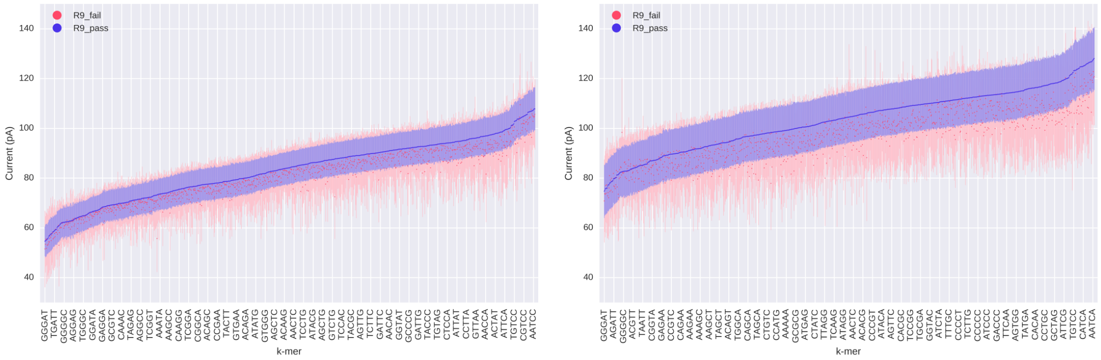

Supplementary Figure 4. Current distributions for experiments with pore version R9 and kit version NSK-007, where the right corresponds to Figure 2d.

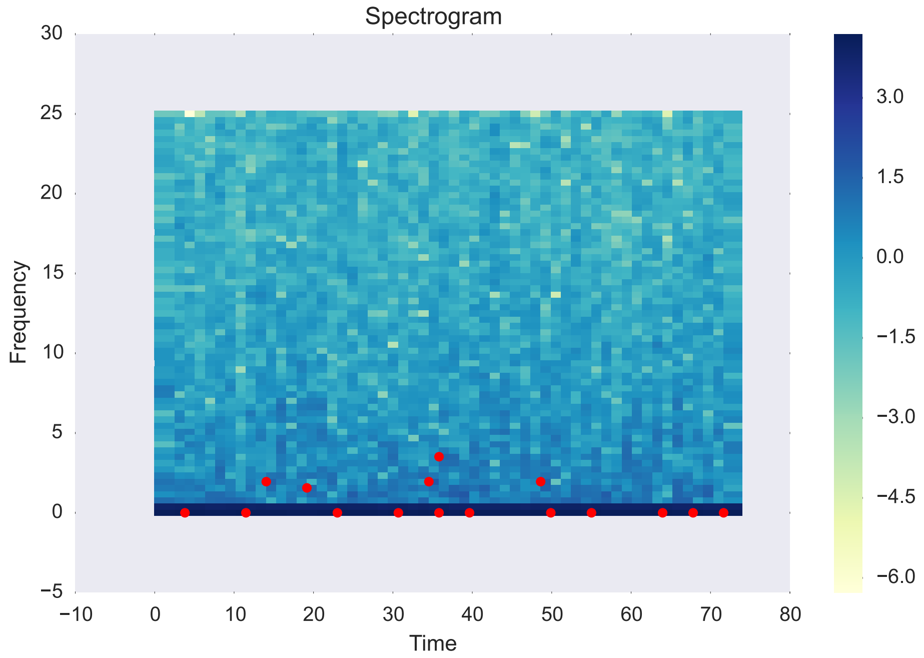

Supplementary Figure 5. Spectrogram of the second flight data read (length = 1752) showing frequencies over time. Parameters used for the fast Fourier transform included a sampling rate of 50, a window size of 128, and an overlap between windows of 64. Peaks with amplitudes over a certain threshold (here, 1.3 on the $\log_{10}$ scale, marked in read) are identified and paired to construct fingerprints.

Flow cell	Total pores	Group 1	Group 2	Group 3	Group 4
1	1440	501	435	351	135
2	1486	509	471	357	149
3	1584	498	476	408	202
4	1483	498	460	349	176
5	1218	471	388	260	99
6	1225	476	400	265	84
7	1261	486	426	263	86
8	1520	509	485	376	150
9	1251	495	426	254	76
10	1060	403	336	233	88
11	1501	509	471	374	147
12	1389	488	426	319	156
13	704	292	219	157	74
14	731	303	228	145	55
15	1374	504	460	304	106
mean	1281.80	462.80	407.13	294.33	118.87
σ^2	269.71	72.10	84.24	78.53	43.28
max	2048	512	512	512	512

Supplementary Table 1. Nanopore flow cell quality control data from flow cells received in 2016. In a first MUX scan, flow cells had an average of 1281.8 available pores. At run time, the four pores surrounding a channel operate sequentially after division into groups.

	Unprotected		Packaged for flight	
Frequency (Hz)	Maximum random vibration (g^2/Hz)	Acceleration (g)	Maximum random vibration (g^2/Hz)	Acceleration (g)
20	0.057	1.07	0.1465	1.71
153	0.057	2.95	0.0002	0.17
190	0.099	4.34	0.0002	0.19
250	0.099	4.97	0.0002	0.22
750	0.055	6.42	0.0002	0.39
2000	0.018	6.00	0.0002	0.63

Supplementary Table 2. Forces experienced during launch vibration by an unprotected payload and a payload packaged for flight inside bubble wrap and a cargo bag.

Test	Available Pores		
	Pre-vibration testing	Post-vibration testing (2/3 dimensions)	Post-vibration testing (3rd dimension)
1	553 (273, 168, 86, 26)	523 (265, 157, 76, 25)	396 (208, 114, 55, 19)
2	538 (279, 161, 78, 20)	517 (272, 153, 69, 23)	386 (221, 101, 47, 17)

Supplementary Table 3. Pore availability before and after 3-axis launch vibration testing. Total pores are listed, with each quartile of channels across the nanopore device shown in parentheses. At each stage (pre-vibration, after axes 1 and 2, and after axis 3), we conducted two MUX scans within a short period. There was not much change between the first and second MUX scans, although the second scan detected fewer available pores in total than the first in all cases. Between the first 2 axes of vibration and the third one, the flow cells were stored at 4°C overnight, which likely contributed to the sharper drop in pore availability in the third set of tests.

Supplementary Video 1 – Demonstration of nanopore sequencing with the MinION^{TM} during parabolic flight.

Supplementary Video 2 – The MinION cover did not easily remain open and had to be restrained in order to load the sample. Aboard the ISS, constant microgravity should ensure that the lid remains as a crewmember places it.
